# Effect of the Norwegian agreement on a more inclusive working life on use of sick leave and pregnancy benefits among pregnant women: a cohort study

**DOI:** 10.1186/s12889-024-20933-8

**Published:** 2024-12-19

**Authors:** Rachel Louise Hasting, Rune Hoff, Suzanne L Merkus, Jon Michael Gran, Ingrid S Mehlum

**Affiliations:** 1 Research Group for Occupational Medicine and Epidemiology, National Institute of Occupational Health, Majorstuen, Oslo, 5330, 0304 PB Norway; 2 Research Group for Work Psychology and Physiology, National Institute of Occupational Health, Oslo, Norway; 3 Oslo Centre for Biostatistics and Epidemiology, Oslo University Hospital, Oslo, Norway; 4 Oslo Centre for Biostatistics and Epidemiology, Department of Biostatistics, University of Oslo, Oslo, Norway; 5 Department of Community Medicine and Global Health, Institute of Health and Society, University of Oslo, Oslo, Norway; 6 Department of Occupational and Environmental Medicine, Copenhagen University Hospital - Bispebjerg and Frederiksberg, Copenhagen, Denmark; 7 Department of Public Health, University of Copenhagen, Copenhagen, Denmark

## Abstract

**Background:**

We aimed to estimate the effect of the voluntary Norwegian Agreement on a More Inclusive Working Life (IA Agreement) on use of sickness absence (SA) and pregnancy benefits among pregnant women.

**Methods:**

Pregnant women (*n* = 112,486) with a birth during 1.12.2003–31.12.2010 were followed from 6 to 37 gestational weeks in a continuous time multistate model with the following states: work, full SA, graded SA, pregnancy benefits, maternity leave, and other. Women working in IA companies were compared to those in non-IA companies regarding incidence and duration of SA and pregnancy benefits. Differences between groups with respect to calendar year, age, civil status, education, industry, and number of employees in the company were adjusted for using inverse probability of treatment weighting. Absolute differences in probabilities over time, expected length of stay (ELOS) in each state and differences in ELOS between IA and non-IA were calculated. 95% confidence intervals (CI) were generated using bootstrapping (1,000 repetitions).

**Results:**

Adjusted analyses suggest that women working in IA companies were more likely to be in full SA in the first and last trimesters, but less likely between 14 and 28 weeks, than those in non-IA companies. The probability of being in work mirrored this, with women in IA companies tending to spend half a day more in work (ELOS difference 0.55, 95% CI -1.79, 3.02). Differences were not statistically significant. The use of graded SA was slightly higher (ELOS difference 0.46, 95% CI -0.87, 1.72) and the use of pregnancy benefits slightly lower (ELOS difference − 0.43, 95% CI -1.32, 0.42) among those in IA companies compared to non-IA companies.

**Conclusions:**

Women in IA companies tended to spend more time in work and graded SA, but less time on pregnancy benefits. Differences in full SA varied during pregnancy and were most positive mid-pregnancy. This indicates that IA measures could be more effective for conditions experienced at this point. However, effects were small and not statistically significant, which may indicate the IA Agreement has not focused much on pregnant women.

**Key terms:**

IA Agreement, MBRN, MoBa, multistate models, pregnancy, pregnancy benefits, sickness absence, work participation.

**Supplementary Information:**

The online version contains supplementary material available at 10.1186/s12889-024-20933-8.

## Background

Around 55,000 live births occur in Norway every year [1]. For the woman giving birth, the 9 months preceding birth can be associated with health problems. Pregnant women between 20 and 44 years had a sickness absence (SA) rate of 21% in 2019, over four times the rate for non-pregnant women of the same age [2]. Many are absent due to pregnancy-related issues, most commonly lumbopelvic pain [3–5]. Though SA can be beneficial for the pregnant woman and the unborn child, long absences from work can also lead to feelings of exclusion and identity loss [3]. Extended SA may also have negative consequences for the employer, who may need to find a temporary replacement and rearrange work tasks if the work requires specialist knowledge. From a societal perspective, increased SA is also costly; the recent budget for Norway estimates that SA will cost the state 64.2 billion Norwegian kroner (approximately €5.2 billion) [6]. It is therefore important that the workplace facilitates pregnant women in participating in work as much as possible, whilst ensuring this is safe for both mother and baby.

Health-related issues during pregnancy can be exacerbated by work exposures, such as standing/walking or night work, resulting in a higher risk of SA [7–9]. In some cases, the exposures may cause harm to the pregnant woman and/or their unborn baby, meaning she should not continue with her usual work duties. One example of this is the use of radiation equipment in radiology [10]. In Norway, women with potentially dangerous working conditions qualify for pregnancy benefits (*svangerskapspenger*). This requires an extensive application, including forms completed by the woman’s doctor/midwife and employer and a declaration that the work tasks cannot be adjusted to avoid the dangerous exposures [11]. These women are not necessarily ill, but they are still absent from work, and thus are also vulnerable to a loss of identity and feeling of exclusion. Employers may vary in the degree to which they assess possibilities for workplace adjustments and in their willingness to adjust work tasks, meaning it may be possible to increase work participation in this group as well as in those at risk for SA. Pregnant women are protected from dismissal or discrimination by their employer through the Working Environment Act [12].

**Fig. 1:**
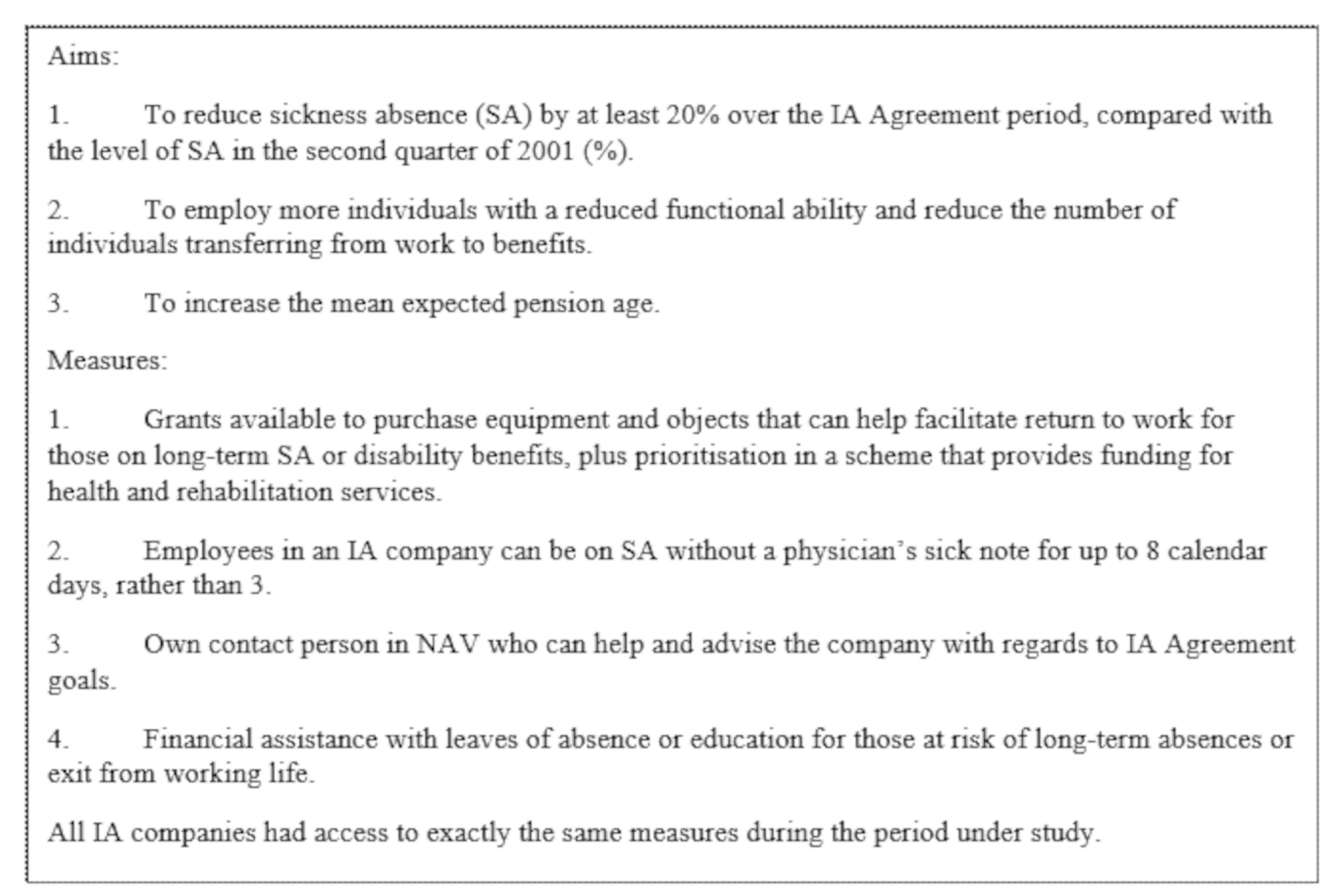
Characteristics of the IA agreement in the study period (2003–2010) [19]

The Norwegian Agreement on a More Inclusive Working Life (the IA Agreement) was introduced in 2001 and aims to increase participation in working life, by for example reducing SA (see Fig. 1) [13]. Companies voluntarily signed the IA Agreement (“IA companies”), pledging to actively work towards the IA Agreement’s goals [13]. This gave them access to measures aimed at reducing both permanent and temporary premature exit from work. These measures included advice and grants for workplace adjustments, accessed through Working Life Centres run by the Norwegian Labour and Welfare Administration (NAV). In 2019, the current IA Agreement and associated measures were expanded to cover all companies [14]. Research on the IA Agreement and SA suggests it could have a small positive effect in reducing SA, though effects are dependent on factors such as gender and industry [15–17]. However, no studies have focused specifically on pregnant women even though they account for around 20% of the gender difference in SA [18]. Evaluating whether the measures included in the IA Agreement could have an effect on SA in pregnant women, could identify whether the large potential to reduce SA is fully utilised among this group.

There is a particular focus on graded (< 100%) SA in the IA Agreement [14], which would allow the pregnant woman to maintain some contact with her workplace and carry out tasks compatible with her work ability. No studies explicitly consider the use of graded SA among pregnant women; we will therefore distinguish between full and graded SA in this study to assess whether the usage differs between IA companies and companies without the IA Agreement (“non-IA companies”).

The aim of this study was therefore to estimate the effect of the IA Agreement on the receipt of graded and full SA, as well as pregnancy benefits among pregnant women. The results can aid both employers and policymakers in understanding whether measures that were part of the IA Agreement were effective for this group, and if the effects differ from previous research on more general populations.

## Methods

### Data sources

The initial population for this study comes from a cohort of all individuals live-born in Norway between 1967 and 1976 (*N* = 626,928). The unique individual identification number was used to link together various registries maintained by Statistics Norway (SSB) and NAV. The “FD-Trygd” events database maintained by SSB [20] was used for the following data: employment dates, SA dates and grade, pregnancy benefit and maternity leave dates, date of baby’s birth and company industry. Data on year and month of mother’s birth (to calculate age at baseline) and civil status were obtained from the National Population Register [21, 22] whilst education information came from the National Education Database (NUDB) [23]: both data sources were accessed via SSB. Information on company size (number of employees) was obtained from the Central Register of Establishments and Enterprises, maintained by SSB [24]. Data on if/when companies entered into the voluntary IA Agreement, changes to their agreement status, and SA diagnoses were obtained from NAV. Data on gestational age at birth, where available, were used from the Medical Birth Registry (MBRN), which is a national health registry containing information about all births in Norway. These data were accessed through the Norwegian Mother, Father and Child Cohort Study (MoBa), a population-based pregnancy cohort study conducted by the Norwegian Institute of Public Health [25]. The current study is based on version 6 of the quality-assured data files released for research in 2011.

Ethical approval was obtained from the Regional Committee for Medical and Health Research Ethics (case number 17344).

### Study design

In this prospective cohort, women were included if they gave birth between 01.12.2003 and 31.12.2010. Pregnancies were followed from 6 weeks gestational age. This was calculated retrospectively either by using available data on gestational age at birth from the MoBa cohort, or when this data was not available, by assuming a full-term pregnancy of 280 days (40 weeks) (see section on Sensitivity Analyses below). Women were followed until 3 weeks before their due date (37 weeks gestational age); this is because the mother is required by law to begin maternity leave by this date and is no longer eligible for sickness benefits [18, 26]. Therefore, the majority of women would be on maternity leave (or have already given birth) by this time point.

### Study population

**Fig. 2:**
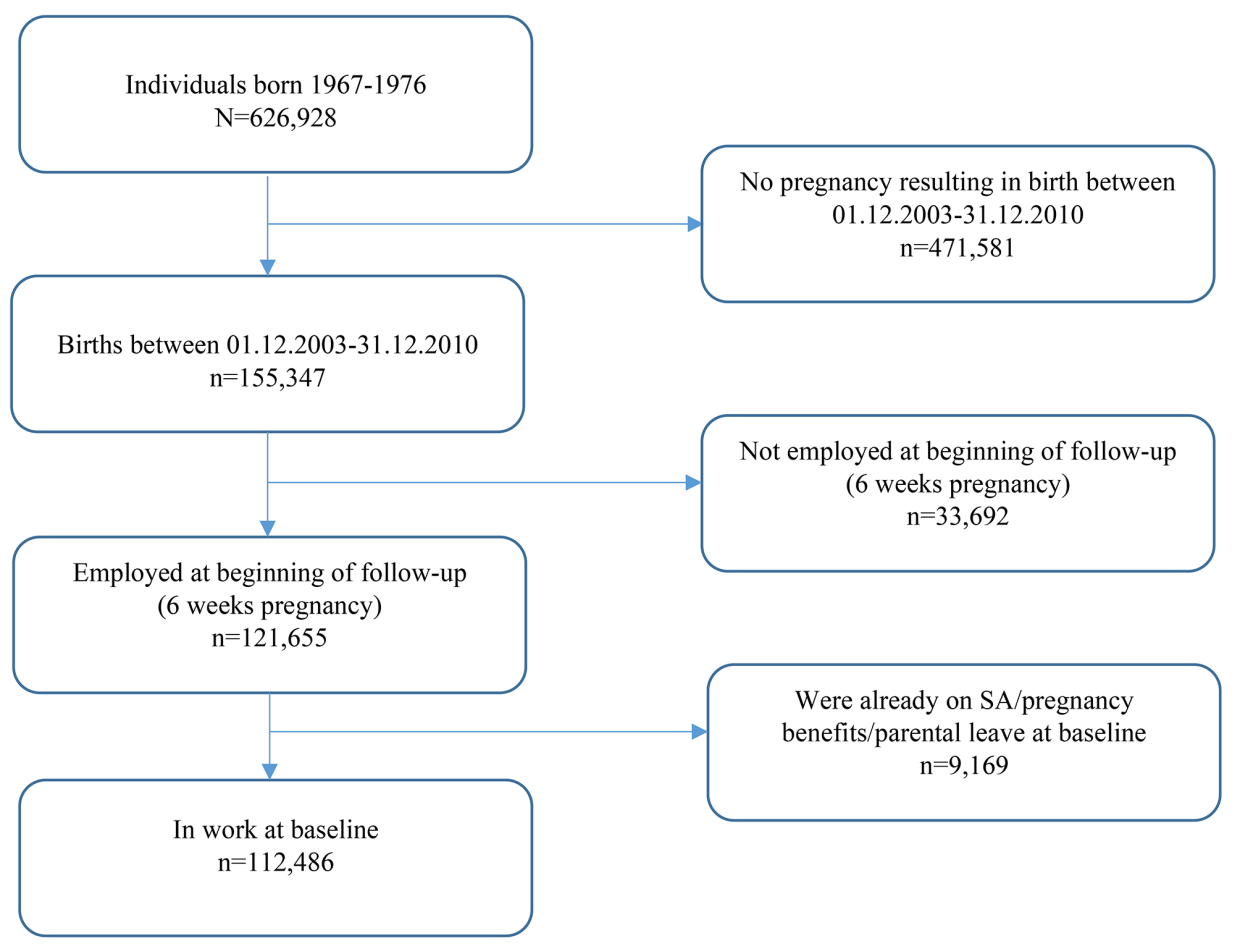
Population flowchart

The initial population consisted of 155,347 pregnancies that resulted in a birth between 01.12.2003 and 31.12.2010 (Fig. 2). Women could contribute with more than one pregnancy. Gestational age data were available for 28,659 pregnancies. Pregnancies were excluded if the woman was not employed at the start of follow-up (*n* = 33,692) or if she was already on SA, pregnancy benefits, or parental leave at 6 weeks gestational age (*n* = 9,169).

### Study outcomes

The first main outcome measures were the differences in the probability of being in work, full SA (100% of contracted working hours), graded SA (< 100%), or pregnancy benefits at each point in time throughout the 31-week follow-up, between women in IA and non-IA companies. The second main outcomes were the differences in expected length of stay (ELOS) in these states, measured in days, between women in IA and non-IA companies over the follow-up period.

In this study, only SA episodes longer than 16 calendar days were included. It is at this point the responsibility for reimbursement passes from the employer to NAV, and the SA episode is registered. The employer can receive reimbursement of this 16 day period if SA is pregnancy-related, so pregnancy-related diagnoses may be registered from day 1 [27]. However, there are no guarantees that all pregnancy-related SA shorter than 16 calendar days is registered. For example, physicians are required to explicitly state that lower back pain is due to pregnancy in the sick note, rather than this happening automatically. Due to this, we have focused on SA > 16 days, in order to assess longer-term SA (rather than short and frequent SA).

### Intervention

The intervention was the IA Agreement, with companies that have chosen to sign the IA Agreement with their local NAV Working Life Centres denoted as “IA companies”. The intervention here is voluntary and not manipulated by the researcher, which can be considered a natural experiment [28]. The IA agreement is signed by companies, not on an individual level. However, given the restriction to women without sick leave at baseline, we can consider the IA Agreement as an intervention that is activated when the need for work facilitation occurs. Under the assumption that this need does not materialise in any notable degree before week 6 of the pregnancy, having an IA Agreement at week 6 will constitute a well-defined intervention.

Pregnancies were grouped according to whether the women worked in IA or non-IA companies at baseline (t = 0, equal to 6 weeks gestational age). IA status was coded as a binary variable (yes/no).

### Covariates

Covariates included in this study were calendar year, age (in years), highest completed education, civil status, industry, and number of employees in the company. All covariates were measured at baseline and were included as potential confounders based on a directed acyclic graph (DAG; see Supplementary Fig. 1). Highest completed education was coded into the following categories: lower secondary education or lower, upper secondary (basic), upper secondary (completed), tertiary (undergraduate), tertiary (graduate). Civil status was coded into a binary variable denoting if they were single (including separated, divorced, or widowed) or married/cohabiting. Industry was coded according to the Standard Industrial Classification 2002 [29], based on the Statistical Classification of Economic Activities in the European Community (NACE) Revision 1.1 prior to 2009, and the NACE Revision 2 in 2009 and 2010. The industry variable included 13 different categories (see Table 1). Where possible, missing values were imputed from either the previous year or the following year (around 2% of the sample); otherwise, values were categorised as missing.

### Statistical analyses

A multistate modelling approach was used, where we analysed successive individual transitions over time between the following states: work, full SA, graded SA, pregnancy benefits, maternity leave, and “other” (not registered in one of the aforementioned states). Note that every individual may experience repeated spells in each state with the exception of the maternity leave state, which was categorised as an absorbing state. The voluntary nature of the IA Agreement means there will be some differences between IA and non-IA companies. To ensure the IA/non-IA groups were balanced with respect to the individuals’ probability of working in an IA company regarding calendar year, age (in years), education, civil status, industry, and number of employees in the company, baseline stabilised inverse probability of treatment weights (sIPTW) were calculated using logistic regression. Unweighted and weighted Nelson-Aalen estimators were used to model transition intensities between states. These intensities were then plugged into the Aalen-Johansen estimator and multiplied with the initial state distribution to calculate state probabilities over time [30], by IA status. We calculated the difference in state probabilities between IA and non-IA companies by subtracting the non-IA state probabilities from the IA state probabilities at each time point. The ELOS was calculated for each group using the area under the state probability curves,, following Eq. (4) in Beyersmann and Putter [30], and we then subtracted the ELOS of non-IA companies from that of IA companies to obtain the ELOS difference. We calculated 95% confidence intervals (CI) using bootstrap with 1,000 repetitions. Data preparation was carried out in Stata, version 16.1 [31]. Analyses were run using R version 3.6.2, partially with use of the package “timereg” [32, 33].

### Sensitivity analyses

We used data from MBRN to identify gestational age at birth for those who were also a part of MoBa; however, this covered only 26% of our sample (*n* = 28,659 pregnancies). We assumed a gestational age at birth of 40 weeks for those not part of MoBa, meaning there will be misclassification of the follow-up end and thus the correct gestational age through follow-up. To assess whether this affected the results, we re-ran the analyses described in the previous section on the subset of individuals present in MoBa.

## Results

The final study population comprised 112,486 pregnancies (72% of the initial population) (Fig. 1). In the unweighted population, women working in IA companies had a slightly higher education than women working in non-IA companies (Table 1). There were a higher number of IA companies in the health/social and education industries, whereas the financial/real estate and wholesale/retail companies had the highest share of non-IA companies. IA companies also tended to have more employees than non-IA companies. Figure 2 shows the observed (unweighted) state probabilities for the full population. The proportion of individuals being in work decreased gradually over the course of the pregnancy, with both full and graded SA most prevalent in the third trimester. The number of events experienced in the IA and non-IA groups are shown in Supplementary Table 1.

**Table 1:** Characteristics of the unweighted study population (*n* = 112,486) at baseline, stratified by IA status

	IA (*n* = 62,064)	Non-IA (*n* = 50,422)
	*n* (%)	*n* (%)
Age (years, mean (SD))	33 (3)	33 (3)
Education		
* Lower secondary or below*	2,155 (3)	3,672 (7)
* Upper secondary*,* basic*	1,737 (3)	2,291 (5)
* Upper secondary*,* completed*	12,308 (20)	17,260 (34)
* Tertiary*,* undergraduate*	36,110 (58)	21,717 (43)
* Tertiary*,* graduate*	9,738 (16)	5,462 (11)
* Missing*	16 (< 1)	20 (< 1)
Civil status		
* Single*	33,709 (54)	29,360 (58)
* Married/in a civil partnership*	28,352 (46)	21,060 (42)
* Missing*	3 (< 1)	2 (< 1)
Industry		
* Agriculture/forestry/fishing*	62 (< 1)	464 (1)
* Mining/quarrying*	648 (1)	592 (1)
* Manufacturing*	3,047 (5)	3,346 (7)
* Electricity/gas/water supply*	206 (< 1)	163 (< 1)
* Construction*	381 (1)	611 (1)
* Wholesale/retail*	2,197 (4)	10,887 (22)
* Hotels/restaurants*	745 (1)	2,031 (4)
* Transport/storage*	1,592 (3)	2,932 (6)
* Financial/real estate*	4,041 (7)	11,513 (23)
* Public administration*	6,132 (10)	2,070 (4)
* Education*	12,707 (20)	2,776 (6)
* Health/social*	28,512 (46)	9,507 (19)
* Other*	1,794 (3)	3,529 (7)
* Missing*	0 (0)	1 (< 1)
Number of employees (mean (SD))	783 (1685)	100 (294)

**Fig. 3:**
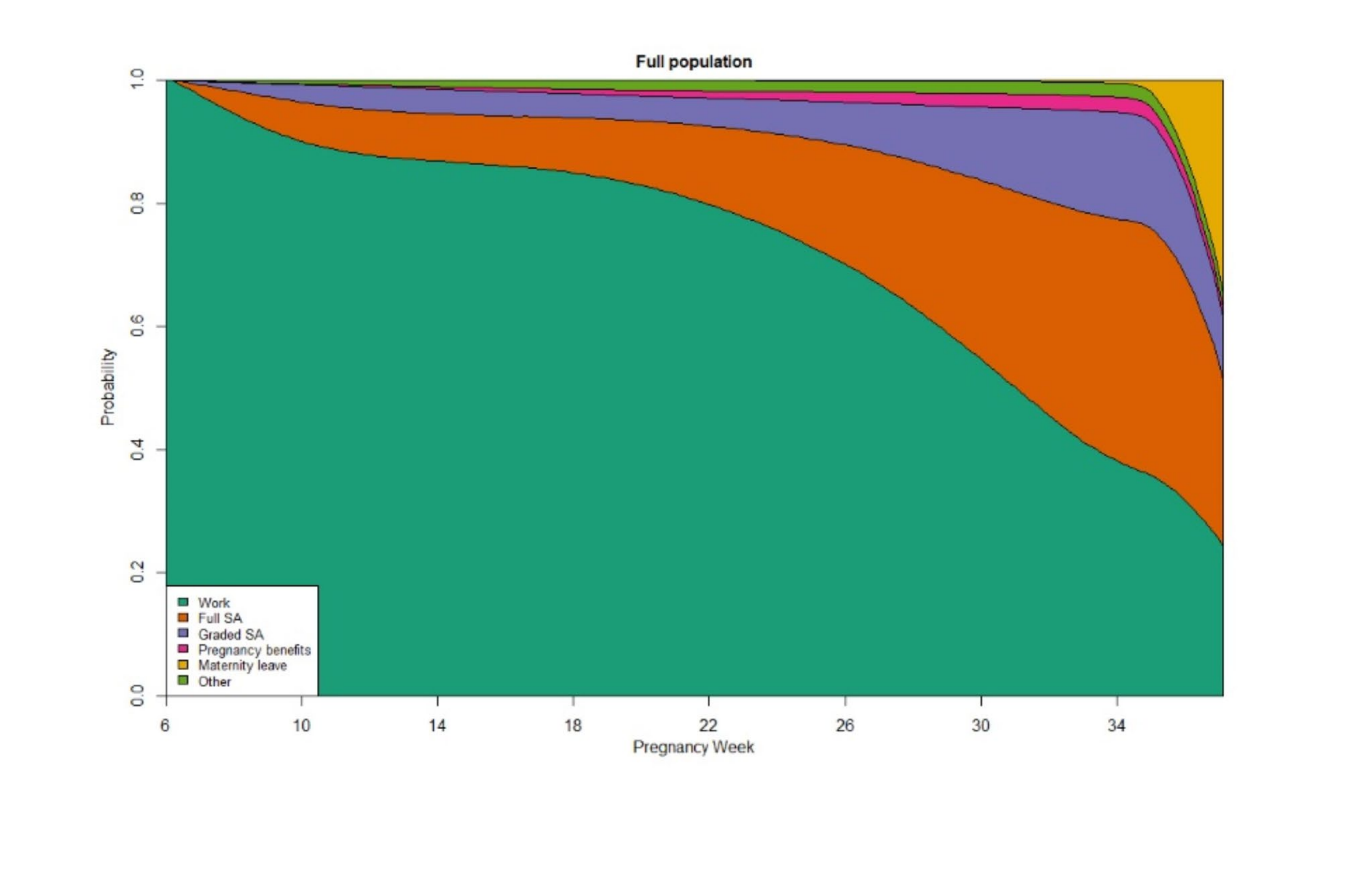
Unweighted state probabilities for the full population

### Main analyses

Differences we found between IA and non-IA companies were small and often the 95% CI included null. However, we identified some interesting trends that we would like to highlight and that could warrant further investigation.

### Full SA

The difference in the probability of being in full SA for women in IA companies compared to non-IA companies varied during the course of the pregnancy, though the 95% CI included null (Fig. 3; see Supplementary Fig. 2 for weighted state probabilities in each group). Women in IA companies had a higher probability of being in full SA from 6 to 14 weeks of pregnancy, peaking at about 10 weeks, which was mirrored by a lower probability of being in work. From 14 to 28 weeks, the probability of being in full SA was marginally lower for women in IA companies compared to women in non-IA companies, before increasing to a higher probability towards the end of follow-up. This pattern is reflected in the ELOS difference between groups, with women in IA companies spending more time in full SA in the first (ELOS difference 0.13 days, 95% CI -0.31, 0.56) and third (ELOS difference 0.53 days, 95% CI -0.53, 1.53) trimesters, but less time in the second trimester (ELOS difference − 0.14 days, 95% CI -0.84, 0.53; Table 2, see Supplementary Table 2 for the ELOS in each group). Throughout the 31-week follow-up period, women in IA companies spent half a day more in full SA than women in non-IA companies (ELOS difference 0.52 days, 95% CI -1.67, 2.61).

**Table 2:** Difference in expected length of stay (ELOS) between individuals in IA and non-IA companies, measured in days, for six work-related states during pregnancy with 95% confidence intervals (CI) calculated using 1,000 bootstrap samples. Numbers presented for weighted whole study population and weighted MoBa subsample. Overall and stratified by trimester

State	Overall ELOS difference, days (95% CI)	Trimester 1 (6–13 weeks) ELOS difference, days (95% CI)	Trimester 2 (14–26 weeks) ELOS difference, days (95% CI)	Trimester 3 (27–37 weeks) ELOS difference, days (95% CI)
*Full population*				
* Work*	0.55 (-1.79, 3.02)	-0.12 (-0.64, 0.41)	0.49 (-0.33, 1.35)	0.19 (-0.82, 1.25)
* Full SA*	0.52 (-1.67, 2.61)	0.13 (-0.31, 0.56)	-0.14 (-0.84, 0.53)	0.53 (-0.53, 1.53)
* Graded SA*	0.46 (-0.87, 1.72)	0.10 (-0.18, 0.37)	0.15 (-0.21, 0.50)	0.21 (-0.48, 0.85)
* Pregnancy Benefits*	-0.43 (-1.32, 0.42)	-0.04 (-0.16, 0.07)	-0.15 (-0.49, 0.17)	-0.24 (-0.68, 0.19)
* Maternity Leave*	-0.09 (-0.33, 0.15)	0.00 (-0.02, 0.02)	0.01 (-0.03, 0.04)	-0.10 (-0.29, 0.09)
* Other*	-1.01 (-1.62, -0.37)	-0.07 (-0.19, 0.06)	-0.35 (-0.57, -0.12)	-0.58 (-0.85, -0.31)
*MoBa subsample*				
* Work*	1.46 (-2.56, 5.41)	0.16 (-0.75, 1.07)	0.55 (-0.80, 1.86)	0.75 (-1.01, 2.47)
* Full SA*	0.30 (-3.16, 3.72)	0.10 (-0.65, 0.82)	0.05 (-1.01, 1.13)	0.15 (-1.50, 1.76)
* Graded SA*	0.57 (-1.89, 3.06)	0.00 (-0.52, 0.52)	0.19 (-0.52, 0.91)	0.37 (-0.86, 1.63)
* Pregnancy Benefits*	-0.46 (-1.52, 0.61)	-0.05 (-0.18, 0.08)	-0.22 (-0.61, 0.15)	-0.19 (-0.73, 0.38)
* Maternity Leave*	-0.06 (-0.34, 0.22)	0.00 (-0.03, 0.03)	0.00 (-0.04, 0.04)	-0.06 (-0.27, 0.15)
* Other*	-1.81 (-2.93, -0.68)	-0.21 (-0.44, 0.02)	-0.58 (-0.97, -0.18)	-1.02 (-1.52, -0.52)

### Graded SA

**Fig. 4:**
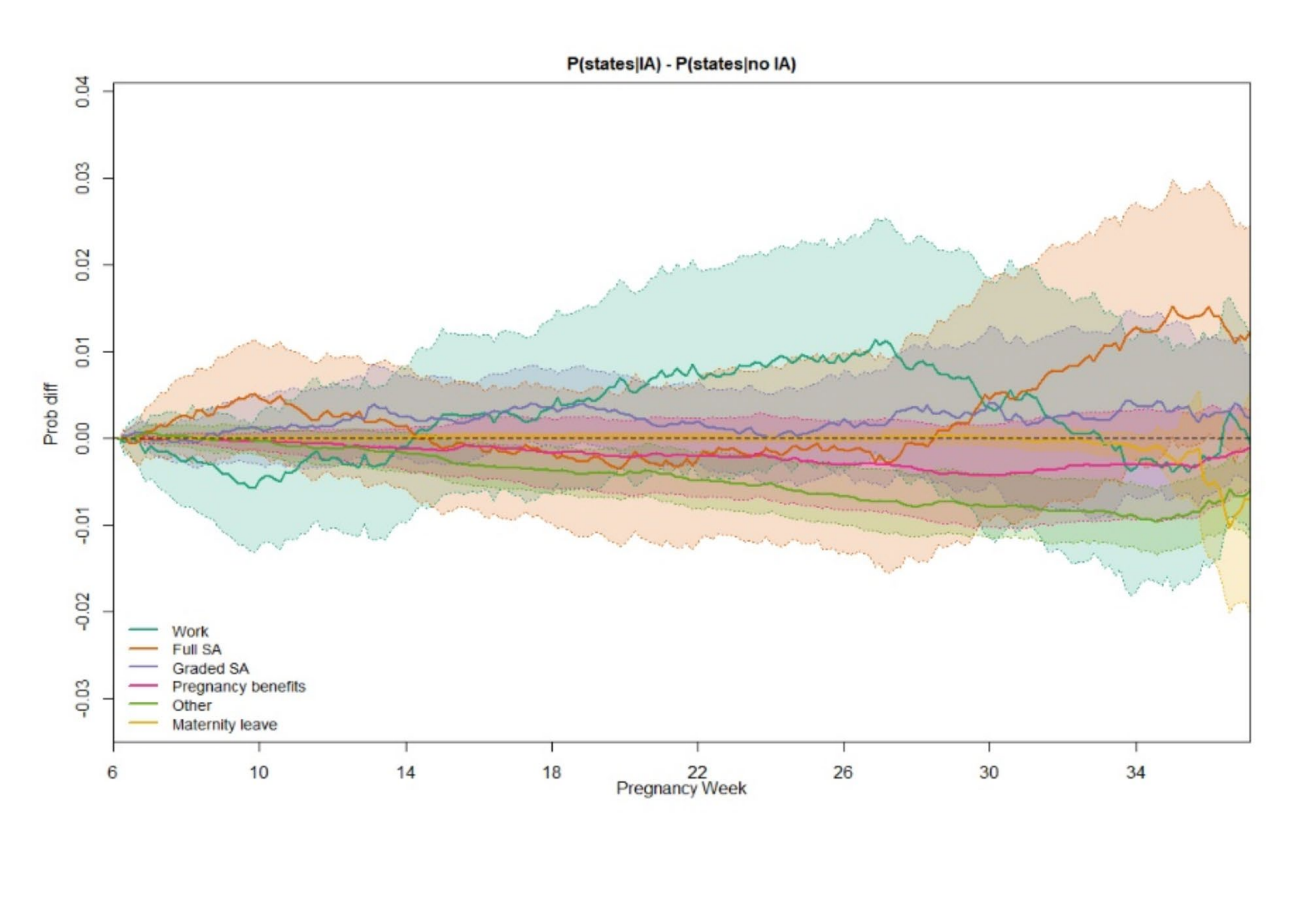
Weighted difference in state probabilities for pregnant women in IA companies compared to those in non-IA companies. 95% confidence intervals calculated using 1,000 bootstrap samples

Women working in IA companies had around a 0.5% points (PP) higher probability of graded SA compared to women in non-IA companies (Fig. 4). This proportion remained fairly constant through the course of the pregnancy and corresponded to just under half a day more in graded SA, though differences were not statistically significant (ELOS difference 0.46 days, 95% CI -0.87, 1.72; Table 2).

### Pregnancy benefits

Women working in IA companies had a marginally lower probability of receiving pregnancy benefits during their pregnancy compared to women in non-IA companies; this was also fairly constant throughout follow-up, though not statistically significant (Fig. 4).

The corresponding ELOS difference was − 0.09 days (95% CI -0.33, 0.15; Table 2).

### Work

The probability of being in work largely mirrored the pattern for full SA, with the 95% CI again including null (Fig. 4). Women in IA companies had a lower probability of being in work up to around 14 weeks of pregnancy, compared to women in non-IA companies. This was followed by a higher probability of being in work up to around 33 weeks, with around a 1% point difference between IA and non-IA women. After 33 weeks, women in IA companies were slightly less likely to be in work compared to women in non-IA companies, though in the third trimester as a whole, women in IA companies spent marginally more time in work in the third trimester (ELOS difference 0.19 days, 95% CI -0.82, 1.25; Table 2). Overall, half a day more was spent in work for women in IA companies (ELOS difference 0.55 days, 95% CI -1.79, 3.02). Differences were not statistically significant.

### Other

Women working in IA companies were less likely to be in the “other” state after about 10 weeks of pregnancy (Fig. 4). This is reflected in the ELOS difference between the groups, with those in IA companies spending over a day less in the “other” state than women in non-IA companies (ELOS difference − 1.01 days, 95% CI -1.62, -0.37; Table 2).

### Sensitivity analyses

**Fig. 5:**
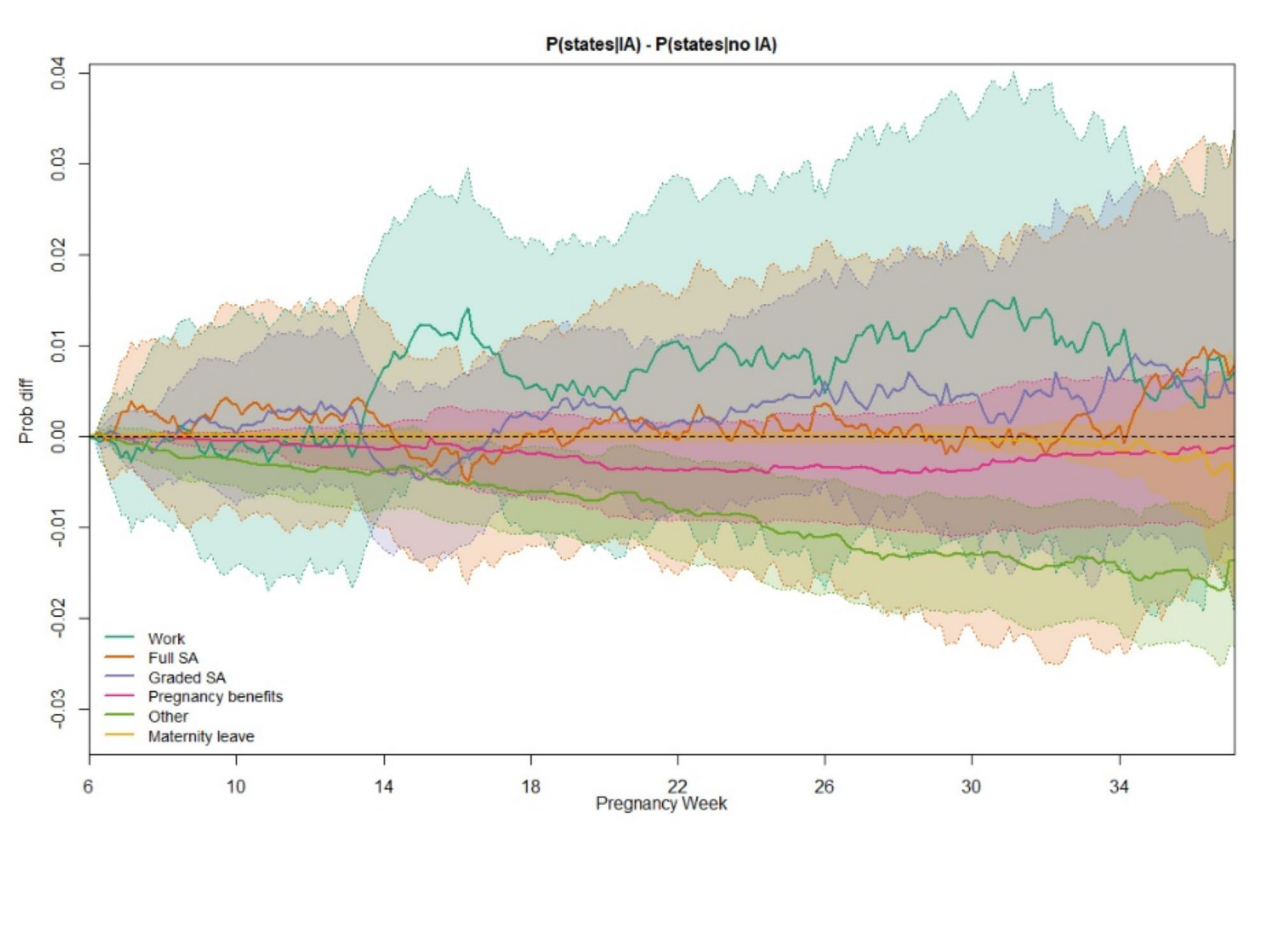
Weighted difference in state probabilities for pregnant women in IA companies compared to those in non-IA companies; only including individuals with MoBa/MBRN data. 95% confidence intervals calculated using 1,000 bootstrap samples

When running the analyses only on women that had information on their due date through MoBa/MBRN data, the results were somewhat different and showed a stronger trend towards a positive impact of the IA Agreement, though results were still non-significant (Fig. 5; Table 2; see also Supplementary Fig. 3 and Supplementary Table 2). The probability of being in work was consistently higher for women in IA companies after 14 weeks, peaking at an ELOS difference of just under 1.5 days compared to women in non-IA companies, though the 95% CI still included null. The ELOS difference in full SA was also marginally lower in the MoBa group than the full population. The exception to this was in the second trimester, where findings were mirrored: individuals in the full population had less time in full SA and the MoBa subgroup had more time in full SA when working in IA companies, compared to non-IA companies. Women in IA companies were also less likely to spend time in the “other” state than women in non-IA companies.

We also ran the same analyses on the MoBa subpopulation with the assumption that all women gave birth in week 40. The results were not significantly different to the main analyses, though the MoBa subpopulation had a slightly higher probability of being in work in the third trimester (Supplementary Figs. 4 & 5).

## Discussion

This study used multistate modelling to study the effects of the IA Agreement on SA and pregnancy benefits in pregnant women. The differences between IA- and non-IA companies were small, with less than a day’s difference between the groups, and the confidence intervals mainly included null. However, we identified some interesting trends that warrant attention. The probability of being in full SA was higher for women in IA companies during the first and last trimesters of pregnancy, but lower in the second trimester. This pattern was largely mirrored by the pattern in probability of being in work. The use of graded SA was in total slightly increased in those working in IA companies. Women working in IA companies spent half a day longer in work, though they were in both full and graded SA half a day longer than those in non-IA companies, respectively. They also spent half a day shorter in pregnancy benefits. The exception to this was the “other” state, which women in IA companies spent less time in compared to women in non-IA companies.

Despite the relatively small effect sizes, our findings may indicate that the IA measures are not as applicable to pregnancy-related conditions as to other causes of reduced work capacity. There may be several reasons for this. The first is that the differences seen may be due to differences between the industries that we could not adjust for in our analyses. We included industry in the sIPTW weights to ensure the proportion of industries were more similar in the IA and non-IA groups. However, this does not adjust for differences within industries and how the effect of the IA Agreement may change depending on which industry is focused on [16, 34]. The industries have also varied with respect to how much they have managed to reduce the overall percentage of SA, with more traditionally male-dominated industries, such as construction and finance/real estate, demonstrating a larger decrease from 2001 to 2017 (20–25%) compared to more female-dominated occupations, such as health/social and education (5–10%) [35]. As the largest share of industries with the IA Agreement in this study were in more female-dominated occupations, this could have attenuated the effect seen here.

Another explanation may be that the SA observed in pregnancy is not necessarily due to objective workplace conditions, but more due to other factors affecting pregnant women’s SA. Some pregnancy-related conditions cannot and should not be adjusted for at work, such as hyperemesis gravidarum (severe morning sickness), threatening miscarriage, or preeclampsia; for such conditions, the IA Agreement would not have any effect and would not be expected to. Another factor concerns the woman’s subjective experience of health and attitudes to SA during a pregnancy compared to other conditions [36, 37]. This can result in higher or lower SA than usual, and can depend on the individual and their physician [37]. In this situation, it would be difficult for the IA measures to have any real effect on individuals’ SA. Finally, pregnancy-related conditions are by their nature time-limited, and the pregnant woman’s need for adjustment may change throughout the course of the pregnancy. This could mean that employers are less motivated to invest in workplace adjustments that may only be needed for a short period of time.

Our results indicate some trends that warrant further attention. For example, the trend seen for work and full SA in women with an IA Agreement relative to those without varied during the pregnancy. Compared to women in non-IA companies, women in IA companies seemed more likely to be in full SA and less likely to be in work during the first and last trimesters of pregnancy, and more likely to be in work and less likely to be in full SA in the second trimester. This indicates that IA measures may be more useful for conditions that are more common in the second trimester, such as lower back pain, leading to the trends we see in this study [15, 16]. As mentioned above, some conditions are difficult to adjust for or should not be adjusted for, such as nausea or preeclampsia, which are more common in the first and third trimesters. Another potential explanation for the trends seen in both the first and third trimester is that IA companies may be more accepting workplaces that make employees feel they can take SA should they need to [37].

There were no major differences in either graded SA or pregnancy benefits between those working in IA and non-IA companies, though women in IA companies were slightly more likely to use graded SA and slightly less likely to use pregnancy benefits. A Swedish study found that almost all women who were not granted access to pregnancy benefits substituted this with SA [36]. The emphasis on dialogue between employees and employers in IA companies when there is a risk of SA, combined with the focus on graded SA, may result in a higher utilisation of graded SA rather than resorting to pregnancy benefits. If it is the case that graded SA is utilised more than pregnancy benefits in IA companies, this would also indicate that they are better at making workplace adjustments, or that the work tasks better allow for adjustments, which would allow the pregnant woman to remain in work.

### Methodological considerations

This study used data from large national registries, which are objectively collected and prevent loss to follow-up. This means that we have a full overview of individuals’ SA and other events. In addition, the use of inverse probability weighting reduced confounder bias, thus increasing the generalisability of our findings, and it allowed for a more causal interpretation of our results, as it balanced the groups with regards to the measured confounders. Despite our usage of sIPTW, it is still possible that some residual confounding is still present. We included industry in our weights, but not specific work exposures, which may differ within the industries and between IA and non-IA companies. Previous research has indicated differences in pregnancy-related SA related to various work exposures [38]. The type of work undertaken and the possibility for adjustments may also influence SA [39]; work in some industries with a larger proportion of IA companies, such as teaching and nursing, are harder to adjust than more sedentary office roles or occupations that do not involve working directly with people.

The study followed only women who were in work at 6 weeks of pregnancy. This means that our results may not be generalisable to all pregnant women. For example, we excluded individuals who were already on SA at 6 weeks, meaning that women who may have a higher rate of SA will not have been included. This may result in an underestimation of the SA rate in pregnant women. This underestimation may not differ significantly between IA and non-IA groups, and thus would not impact the differences seen in this study. However, if IA companies are better at reducing the duration of SA and including individuals with a higher risk of SA in the workplace [15, 16], pregnant women in IA companies may appear to have a higher rate of SA than those in non-IA companies during follow-up. This is because women in IA companies would then be more likely to be registered as in work at inclusion than similar individuals in non-IA companies. We were also not able to include immigrant women, as the data material includes only individuals who were born in Norway. There is a possibility that immigrant women would be more likely to work in sectors with a lower share of IA companies and may in addition have work tasks that cause or exacerbate pregnancy-related issues (e.g., cleaners and shop workers, who have many manual tasks). The results of this study are likely not representative of this group.

Finally, we did not have available information on how far through pregnancy women were when they gave birth for around three quarters of the sample. We used instead an estimation of 280 days, which is the due date used by the healthcare services. Only a very small number of women actually give birth on their due date, so our assumption is likely wrong; in addition, there may be women in our sample who gave birth prematurely (i.e., before 37 weeks). We have no way of identifying these individuals from our data, which means some individuals are likely followed up from before pregnancy. As SA tends to be much higher in pregnancy, this would lead to an underestimation of SA in our study, along with a misclassification of SA; however, we can consider this to be non-differential misclassification as the lack of due date data was not dependent on IA status. The risk of this having an impact on the results is likely small, however, as only 4% of women in the MoBa subgroup had given birth before 37 weeks and sensitivity analyses assuming all in the MoBa subsample gave birth in week 40 did not significantly change the results, though they had a slightly higher probability of being in work in the third trimester (Supplementary Figs. 4 and 5). Our sensitivity analyses using the MoBa subgroup with correct due date information showed some changes compared with the main results, including a higher probability for women in IA companies to be in work after the first trimester of pregnancy and a more similar probability of full SA between IA and non-IA companies. This could indicate that substantial misclassification of follow-up start occurred in the women not part of MoBa, though the direction of the results did not change. This could also mean that if we had information on gestational age at birth in the whole population, a larger effect of the IA Agreement may have been found. There are some differences between the two populations indicating selection bias in the MoBa population; the women in our MoBa subgroup were more highly educated regardless of group, for example. Other research has also indicated selection bias in MoBa participation [40]. The differences in results may, therefore, also be partly a result of this bias rather than only due to a misdefinition of follow-up start.

### Implications and future research

This is one of the first studies to look at work participation in pregnant women with the use of more advanced modelling techniques for individual multi-state transitions. We found that women employed in IA companies tended to have on average half a day more in full SA; however, they also tended to have half a day more in work during the follow-up period compared to women in non-IA companies. If this difference can be assigned to the IA Agreement, it corresponds to a small effect on the individual level, but over 30,000 more working days gained on a population level just for those working in IA companies in our study (60,494 women over a span of 7 years). Our findings imply that the IA Agreement could be effective at keeping women in work during the second trimester of pregnancy, despite the small effect sizes and the higher rate of SA in IA companies. As the current IA Agreement covers all companies [14], the potential for increasing work participation among pregnant women is large. As work tasks and the ability to adjust these also differ between industries and occupations, further work could look closer at different subgroups within the working population to see where the IA Agreement may be most effective for pregnant women.

The intervention in this study was whether the individual had worked in a company that signed a local IA Agreement with their NAV Working Life Centre. We did not have the opportunity to investigate here how much a company used the IA measures, or whether the women required workplace adjustments and if so, whether they were given. One study using the same MoBa cohort studied the associations between adjustments and probability of SA and return to work, finding that women who required and received job adjustment had a reduced risk of SA and increased probability of return to work [41], but this was on a more general level and not related to the IA Agreement. Further research focusing on specific IA measures could help with answering this question.

We only considered SA that was longer than 16 calendar days in this study. It may be the case that different effects of the IA Agreement on both the amount of SA and the differences between the groups would have been seen if data on shorter periods of absence had been available. However, the data sources available in this study are likely incomplete for shorter absence periods; further research with different data sources could further investigate IA Agreement effects on shorter periods of absence among pregnant women. The change in absolute probability of full SA and of being in work throughout the course of the pregnancy for those in IA and non-IA companies also warrants further exploration into the intricacies of workplace adjustments during pregnancy, and the effect of implemented measures that are not necessarily developed with pregnancy in mind.

## Conclusions

In this study, pregnant women working in a company that had signed a local IA Agreement tended to spend more time in work and graded SA (over 16 calendar days), but less time on pregnancy benefits. The probability of being in full SA (over 16 calendar days) varied depending on trimester, with a lower probability in the second trimester for women in IA companies. Women in IA companies also spent on average half a day longer in full and graded SA, respectively, but also had half a day longer in work over the follow-up period compared to those in non-IA companies. Finally, women in IA companies spent half a day shorter on pregnancy benefits than those in non-IA companies. However, the effect sizes were small, and the confidence intervals included the null. The trends observed in this study do not rule out that there could be significant differences between industries, which should be investigated further.

## Electronic supplementary material

Below is the link to the electronic supplementary material.

Supplementary Material 1

## Data Availability

The data that support the findings of this study are available from Statistics Norway (FD-Trygd/Central Register of Establishments and Enterprises/Central Population Register/National Education Database) and the Norwegian Labour and Welfare Administration (NAV; IA Agreement data) and were collected in accordance with national guidelines. Restrictions apply to the availability of these data, which were used under license for the current study, and so are not publicly available. Contact the corresponding author for more details on data availability. Data from the Norwegian Mother, Father and Child Cohort Study and the Medical Birth Registry of Norway used in this study are managed by the national health register holders in Norway (Norwegian Institute of public health) and can be made available to researchers, provided approval from the Regional Committees for Medical and Health Research Ethics (REC), compliance with the EU General Data Protection Regulation (GDPR) and approval from the data owners. The consent given by the participants does not open for storage of data on an individual level in repositories or journals. Researchers who want access to data sets for replication should apply through helsedata.no. Access to data sets requires approval from The Regional Committee for Medical and Health Research Ethics in Norway and an agreement with MoBa.

## Abbreviations

CI: Confidence interval
DAG: Directed acyclic graph
ELOS: Expected length of stay
IA Agreement: Agreement on a More Inclusive Working Life
MBRN: Medical Birth Registry of Norway
MoBa: Norwegian Mother, Father and Child Cohort Study
NACE: Statistical Classification of Economic Activities in the European Community
NAV: Norwegian Labour and Welfare Administration
NUDB: National Education Database
SA: Sickness absence
sIPTW: Stabilised inverse probability of treatment weights
SSB: Statistics Norway
