# Supplementary material for: Effect of the Norwegian agreement on a more inclusive working life on use of sick leave and pregnancy benefits among pregnant women: a cohort study
Source: BMC Public Health. 2024 Dec 19;24:3536. doi: 10.1186/s12889-024-20933-8 (PMC11660774; doi:10.1186/s12889-024-20933-8)
Supplement: Supplementary file 1 — Supplementary Material 1 [file 12889_2024_20933_MOESM1_ESM.docx]

**Supplementary Material**

**
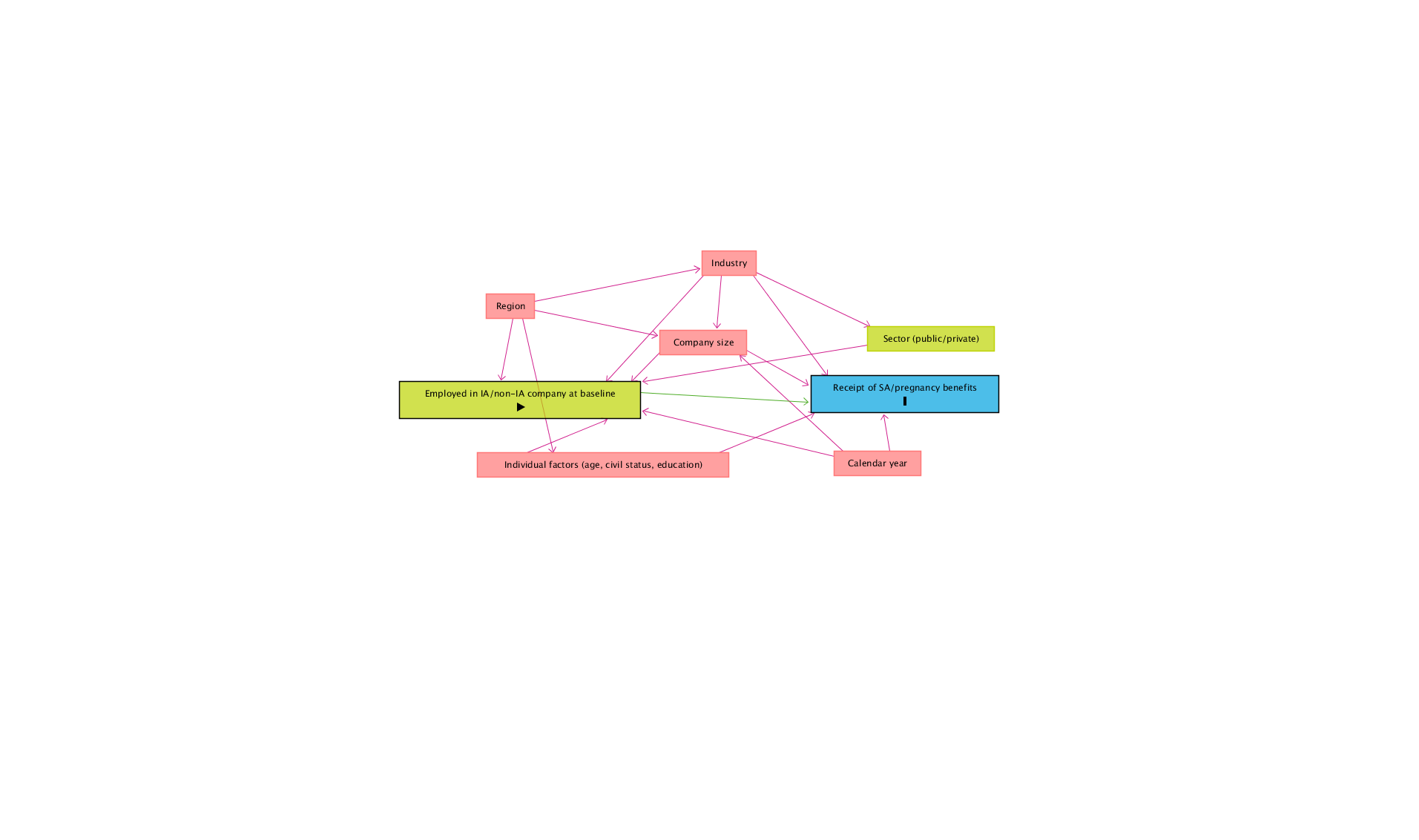
**

**Supplementary Figure 1.** Directed acyclic graph (DAG) for the effect of being employed in an IA company at baseline on receipt of SA/pregnancy benefits

**
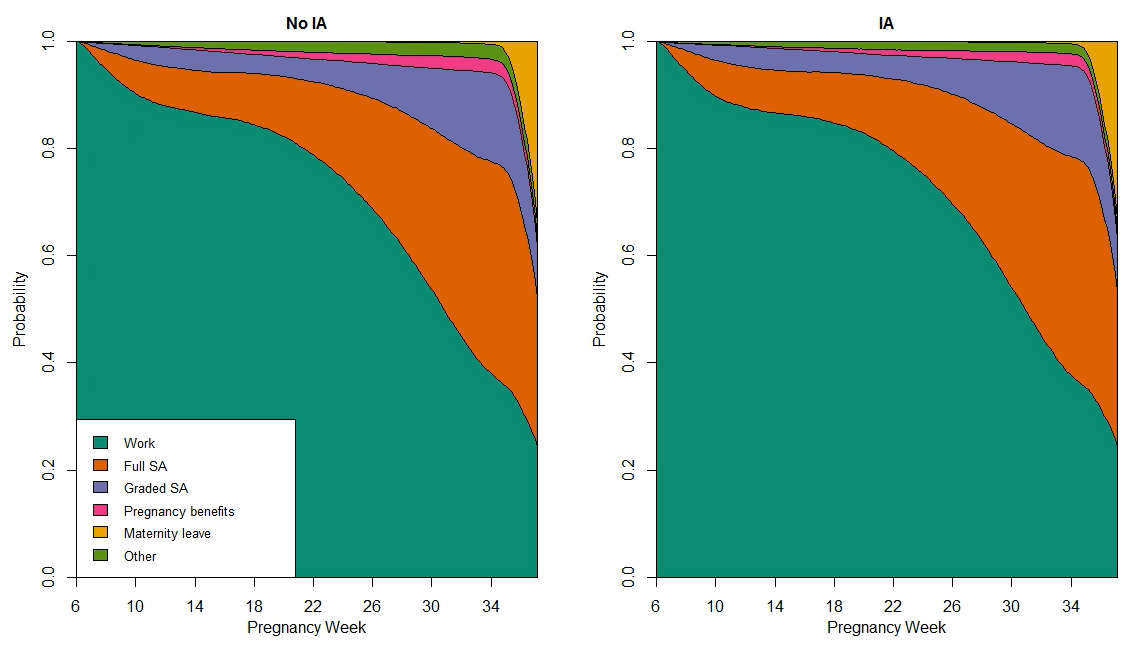
**

**Supplementary Figure 2.** Weighted state probabilities for pregnant women in non-IA and IA companies.


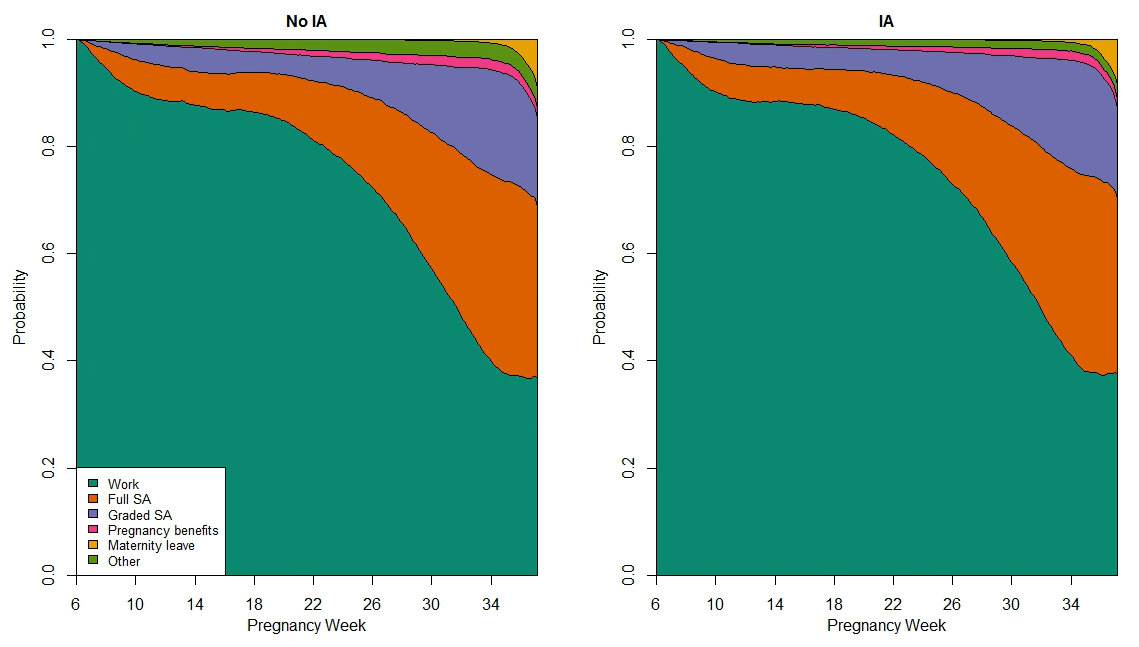

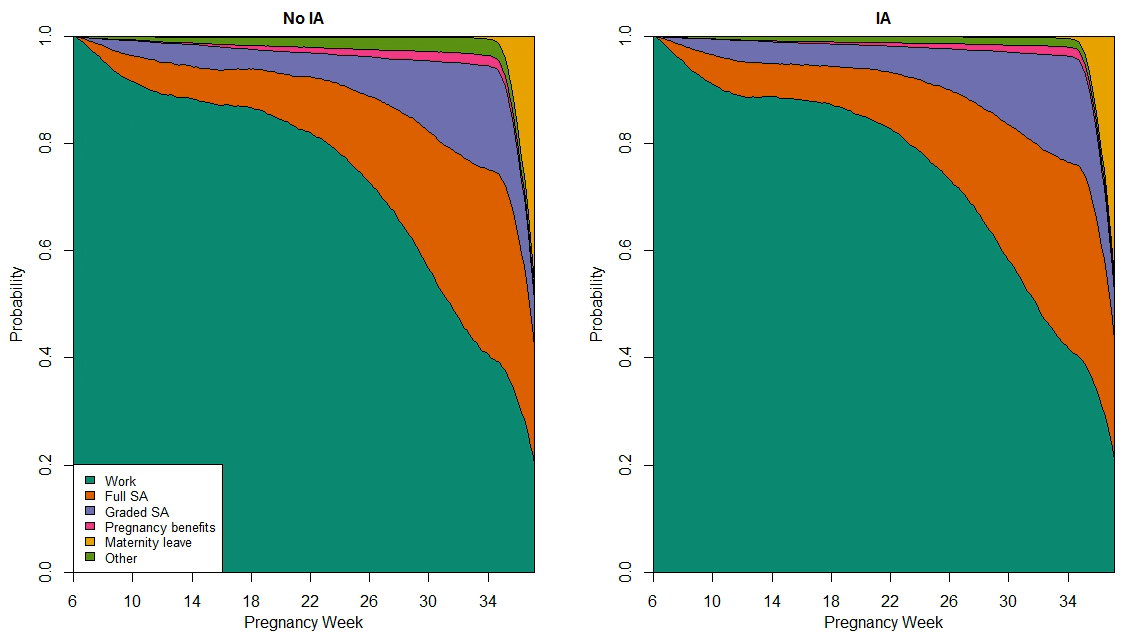


**Supplementary Figure 3.** Weighted state probabilities for pregnant women in non-IA and IA companies, only including individuals with MoBa/MBRN data.

**Supplementary Figure 4.** Weighted state probabilities for pregnant women in non-IA and IA companies. Only including individuals with MoBa/MBRN data, with an.additional assumption that women gave birth at 40 weeks gestational age.


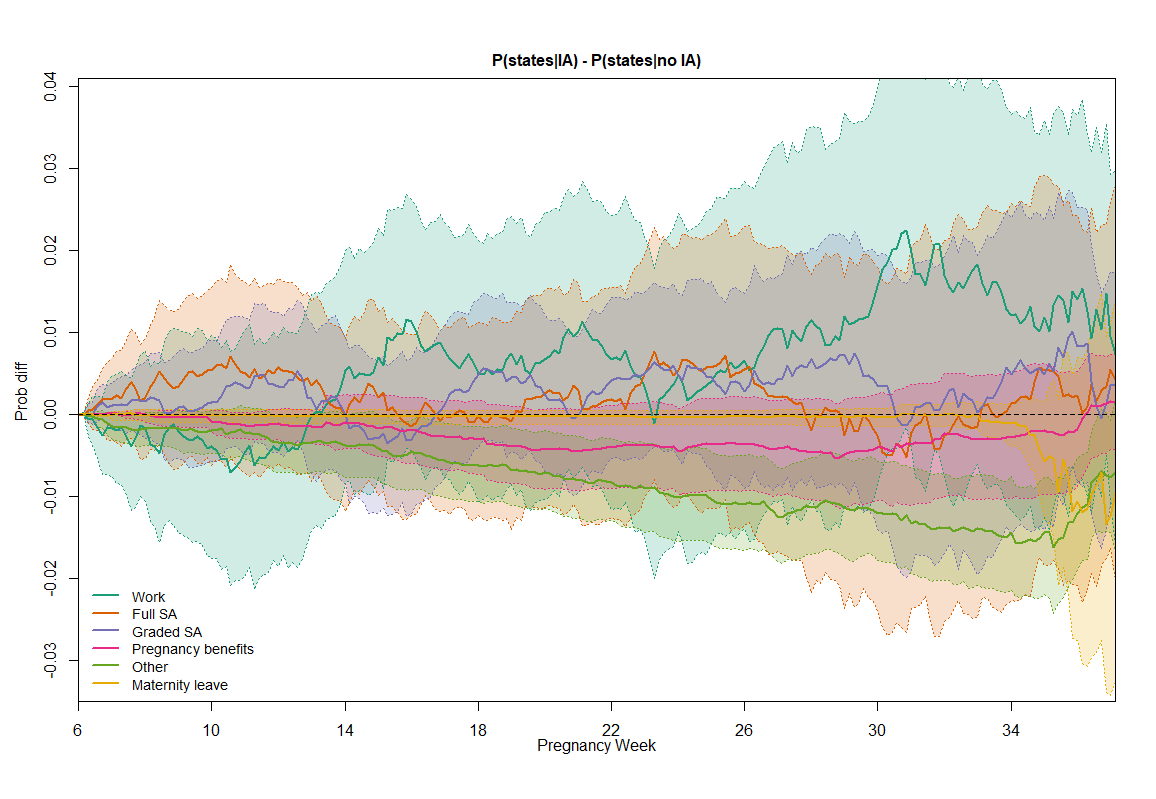


**Supplementary Figure 5.** Weighted difference in state probabilities for pregnant women in IA companies compared to those in non-IA companies; only including individuals with MoBa/MBRN data, with additional assumption that women gave birth at 40 weeks gestational age. 95% confidence intervals calculated using 1,000 bootstrap samples.

| **Supplementary Table 1.** Number of events recorded during follow-up period in weighted main study population (n=112,490) and the weighted subgroup with MoBa data (n=28,522). Note that individuals can have no events or multiple events during follow-up. | | |
| --- | --- | --- |
| State | IA | Non-IA |
| Full population | n=63,454 | n=49,036 |
| *(Return to) Work* | 21,803 | 15,923 |
| *Full SA* | 36,461 | 26,951 |
| *Graded SA* | 18,417 | 13,746 |
| *Pregnancy Benefits* | 1,795 | 1,545 |
| *Maternity Leave* | 22,079 | 17,455 |
| *Other* | 3,766 | 3,571 |
|  |  |  |
| MoBa subgroup | n=16,408 | n=12,114 |
| *(Return to) Work* | 5,239 | 3,802 |
| *Full SA* | 8,321 | 6,014 |
| *Graded SA* | 5,469 | 3,939 |
| *Pregnancy Benefits* | 371 | 291 |
| *Maternity Leave* | 1,513 | 1,183 |
| *Other* | 708 | 808 |

| **Supplementary Table 2.** Expected length of stay (ELOS) in days for six work-related states during pregnancy with 95% confidence intervals (CI) calculated using 1,000 bootstrap samples. Numbers presented for weighted whole study population and weighted MoBa subsample. Stratified by IA status and trimester. | | | | | | |
| --- | --- | --- | --- | --- | --- | --- |
|  | Trimester 1  6-13 weeks | | Trimester 2  14-26 weeks | | Trimester 3  27-37 weeks | |
| State | IA  ELOS in days  (95% CI) | Non-IA  ELOS in days  (95% CI) | IA  ELOS in days (95% CI) | Non-IA  ELOS in days (95% CI) | IA  ELOS in days (95% CI) | Non-IA  ELOS in days (95% CI) |
| *Full population* |  |  |  |  |  |  |
| *Work* | 65.89  (65.65, 66.16) | 66.02  (65.74, 66.28) | 57.74  (57.37, 58.15) | 57.26  (56.80, 57.70) | 33.71  (33.24, 34.24) | 33.52  (32.99, 34.06) |
| *Full SA* | 4.46  (4.25, 4.67) | 4.33  (4.11, 4.56) | 10.01  (9.67, 10.32) | 10.14  (9.80, 10.51) | 24.60  (24.10, 25.09) | 24.08  (23.56, 24.63) |
| *Graded SA* | 2.06  (1.92, 2.18) | 1.95  (1.82, 2.10) | 3.46  (3.28, 3.63) | 3.31  (3.13, 3.49) | 9.52  (9.22, 9.81) | 9.31  (8.95, 9.69) |
| *Pregnancy Benefits* | 0.14  (0.10, 0.19) | 0.19  (0.12, 0.26) | 0.74  (0.61, 0.87) | 0.89  (0.70, 1.09) | 1.44  (1.27, 1.62) | 1.68  (1.43, 1.95) |
| *Maternity Leave* | 0.02  (0.01, 0.03) | 0.02  (0.01, 0.02) | 0.03  (0.02, 0.06) | 0.03  (0.02, 0.04) | 2.38  (2.28, 2.48) | 2.47  (2.38, 2.57) |
| *Other* | 0.43  (0.38, 0.50) | 0.50  (0.44, 0.57) | 1.02  (0.93, 1.13) | 1.37  (1.26, 1.50) | 1.35  (1.24, 1.48) | 1.94  (1.79, 2.09) |
| *MoBa subsample* |  |  |  |  |  |  |
| *Work* | 66.48  (66.05, 66.92) | 66.32  (65.84, 66.80) | 59.71  (59.09, 60.34) | 59.15  (58.48, 59.89) | 37.09  (36.29, 37.95) | 36.35  (35.48, 37.30) |
| *Full SA* | 3.90  (3.55, 4.24) | 3.80  (3.41, 4.20) | 8.19  (7.69, 8.72) | 8.14  (7.58, 8.70) | 21.42  (20.65, 22.19) | 21.27  (20.43, 22.15) |
| *Graded SA* | 2.17  (1.93, 2.41) | 2.17  (1.90, 2.45) | 3.75  (3.42, 4.09) | 3.56  (3.18, 3.94) | 11.48  (10.93, 12.04) | 11.11  (10.41, 11.78) |
| *Pregnancy Benefits* | 0.09  (0.04, 0.14) | 0.14  (0.07, 0.22) | 0.50  (0.35, 0.67) | 0.72  (0.52, 0.96) | 1.13  (0.89, 1.39) | 1.32  (1.01, 1.62) |
| *Maternity Leave* | 0.02  (0.00, 0.03) | 0.01  (0.00, 0.03) | 0.02  (0.01, 0.04) | 0.02  (0.01, 0.05) | 0.69  (0.60, 0.79) | 0.76  (0.64, 0.87) |
| *Other* | 0.35  (0.26, 0.45) | 0.56  (0.43, 0.70) | 0.83  (0.68, 0.99) | 1.41  (1.17, 1.65) | 1.18  (0.99, 1.38) | 2.20  (1.90, 2.51) |
